# Case report: dysgeusia, strawberry tongue, and psoriatic eruptions after combination treatment with adalimumab, sulfasalazine, and etoricoxib for ankylosing spondylitis

**DOI:** 10.3389/fmed.2025.1419922

**Published:** 2025-02-03

**Authors:** Binglin Cui, Jing Lin, Yuanchun Huang, Huachen Zhu, Jinbo Zou, Jiesheng Qin, Hui Pan, Jian Chen

**Affiliations:** ^1^Department of Pediatrics, The First Affiliated Hospital of Shantou University Medical College, Shantou, Guangdong, China; ^2^Department of Oncology, The First Affiliated Hospital of Shantou University Medical College, Shantou, Guangdong, China; ^3^Microbiology Division, Department of Clinical Microbiology, The First Affiliated Hospital of Shantou University Medical College, Shantou, Guangdong, China; ^4^Guangdong-Hong Kong Joint Laboratory of Emerging Infectious Diseases, Joint Institute of Virology (STU/HKU), Shantou University, Shantou, Guangdong, China; ^5^Joint Laboratory for International Collaboration in Virology and Emerging Infectious Diseases, Joint Institute of Virology (STU/HKU), Shantou University, Shantou, Guangdong, China; ^6^State Key Laboratory of Emerging Infectious Diseases, Li Ka Shing Faculty of Medicine, School of Public Health, The University of Hong Kong, Hong Kong, Hong Kong SAR, China; ^7^Department of Dermatology, The First Affiliated Hospital of Shantou University Medical College, Shantou, Guangdong, China; ^8^Department of Otorhinolaryngology, The First Affiliated Hospital of Shantou University Medical College, Shantou, Guangdong, China; ^9^Department of Outpatient, Shantou Longhu People’s Hospital, Shantou, Guangdong, China; ^10^Clinical Research Unit, Shantou University Medical College, Shantou, Guangdong, China; ^11^Department of Neurosurgery, The First Affiliated Hospital of Shantou University Medical College, Shantou, Guangdong, China

**Keywords:** dysgeusia, strawberry tongue, psoriasis-like eruption, adalimumab, adverse drug reaction, case report

## Abstract

**Background:**

Tumor necrosis factor blockers can suppress immune system and lead to various adverse drug reactions, including deaths. We present a rare case of dysgeusia, strawberry tongue, and psoriasis-like eruptions after simultaneous administration of adalimumab, sulfasalazine, and etoricoxib.

**Case description:**

A 36 year-old male with ankylosing spondylitis presented with progressive loss of taste for 3 months and rashes on his upper trunk for 5 days. He had been receiving adalimumab, sulfasalazine, and etoricoxib for 9 months. After self-discontinuing the medicines, the rashes gradually subsided. On examination, swollen and red strawberry tongue were noticed. *Acinetobacter pittii* was isolated by sputum culture. The patient refused any treatment. During follow-up, recurrent dispersed papules/macules and desquamation appeared on his upper trunk, with erythema and erosion in umbilical region. Subsequently, generalized scalp erythema, exudates, scabs, hair bundles, redness and desquamation behind ears occurred. The tongue, taste, and skin lesions resolved sequentially and steadily until complete recovery.

**Outcome:**

He remained in remission during 4 years follow-up. The total course of disease was around 10 months.

**Discussion:**

Clinicians should be cautious of the adverse drug reactions/events due to adalimumab, sulfasalazine, and etoricoxib. Rational use of medicines is advocated.

## 1 Introduction

Tumor necrosis factor (TNF) blockers, comprising etanercept, infliximab, adalimumab, certolizumab pegol, and golimumab, have been well-recognized as one of the most promising therapies for various inflammatory diseases, including ankylosing spondylitis. Patients receiving TNF blocker regimen which inhibits immune system are at increased risk of diversified adverse drug reactions or events (including rashes, severe infections, and even deaths), especially when taking concomitant immunosuppressants (1–3). Herein, we report a unique case of dysgeusia, strawberry tongue, and psoriasis-like eruptions after simultaneous administration of adalimumab, sulfasalazine, and etoricoxib for ankylosing spondylitis.

## 2 Case presentation

**FIGURE 1:**
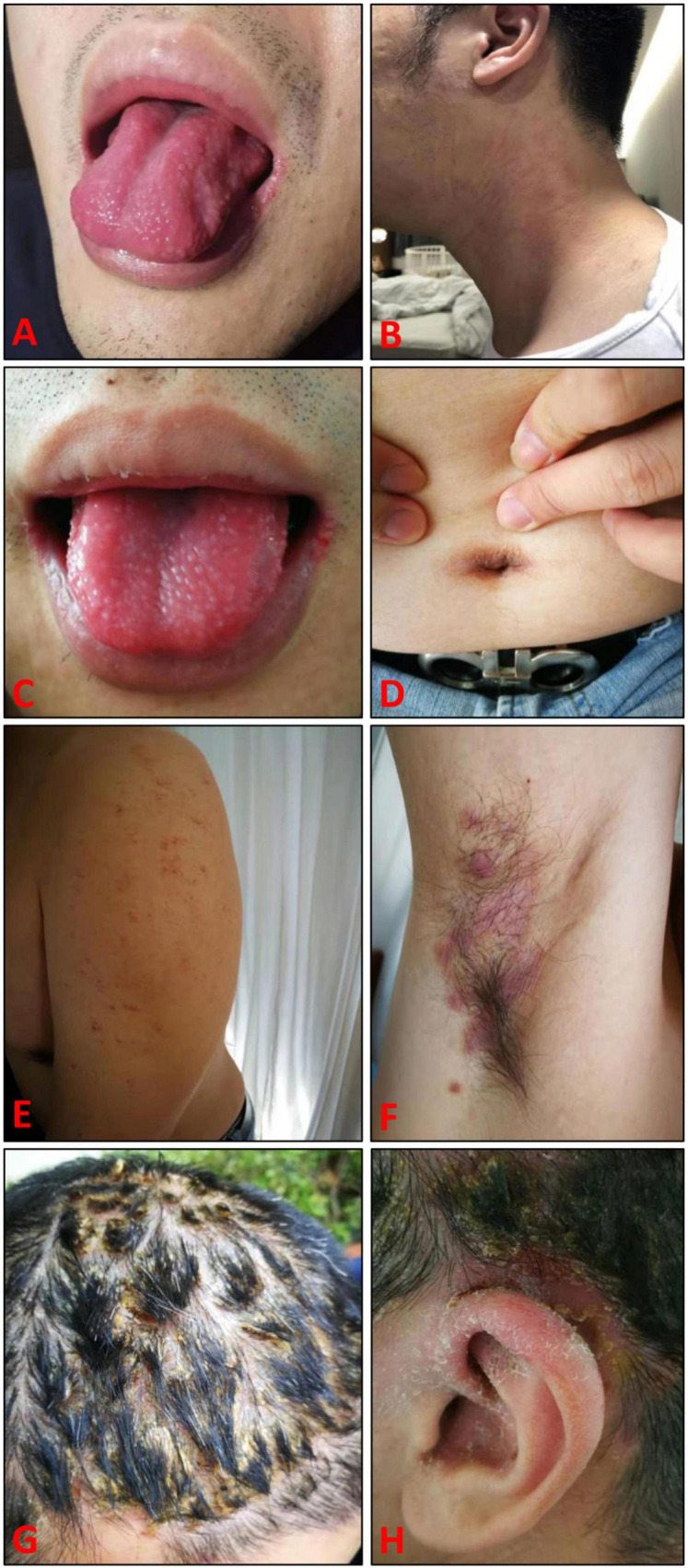
Clinical manifestations of adverse drug events caused by combination therapy with adalimumab, sulfasalazine, and etoricoxib for ankylosing spondylitis. **(A)** Strawberry tongue; **(B)** Small red maculae and maculopapular scattering on the neck; **(C)** Swollen and red strawberry tongue with visible teeth marks on the edge; **(D)** Erythema and erosion in umbilical region; **(E,F)** Dispersed papules/macules and desquamation in armpit and on upper arm; **(G)** Generalized scalp erythema, exudates, scabs, hair bundles, redness and desquamation behind ears; **(H)** Scalp pustules, desquamation, and fissures, in addition to dryness and desquamation around ear.

A 36 year-old Chinese male presented with aggravating loss of taste for 3 months and rashes on his upper trunk for 5 days at Shantou Longhu People’s Hospital in September 2019. He was diagnosed with ankylosing spondylitis for 7 years spinal pain in October 2018 and thereafter was initiated on therapy with subcutaneous injection of adalimumab (40 mg every 3 weeks), oral sulfasalazine (1 g twice per day), and oral etoricoxib (60 mg every 2 days) after careful exclusion of any pre-existent infections. Decreased saliva, dry lips, tongue numbness, and loss of taste appeared in June 2019; blisters occurred on the dorsum of swollen tongue in July 2019 (Figure 1). His tongue became painful and more swollen, and small red maculae and maculopapular scattered around his upper trunk 5 days before the visit (Figure 1). After self-discontinuation of the medicines, the pain and swelling of tongue were alleviated noticeably despite of no apparent change in his taste; the rashes gradually subsided, and his fingers were desquamated bilaterally. From the onset of illness, he occasionally had productive cough of yellow sputum and night sweats without fever/chills. He denied any histories of hepatitis, tuberculosis, sexually transmitted diseases, food/medicine allergies, or recent intake of other medications, but had occasional alcohol consumption and cigarette smoking. His brother had ankylosing spondylitis as well but rejected any treatment. Other family members were generally healthy.

On examination, dry lips, swollen and red strawberry tongue with visible teeth marks on the edge were noticed (Figure 1); submandibular and cervical lymph nodes were enlarged but non-tender to palpation. Other physical examinations were unremarkable.

His main laboratory results are summarized in Supplementary Material 1. Very low titer of antibodies to dust mite (0.43; reference range: < 0.35) was detected in a 19-item screening test for allergens. Cervical ultrasound showed bilateral and multiple hypoechoic nodules, indicating reactive lymphadenopathy. Chest X-ray and ultrasound on heart, liver, gallbladder, pancreas, spleen, and urinary system were basically normal. *Acinetobacter pittii* was isolated by sputum culture. No pathogenic pathogens (i.e., normal flora) were found through wide-spectrum gene sequencings from tongue coating, sputum, saliva, and urine specimens. Nasopharyngoscopy revealed diffused swellings of tongue root, epiglottis, laryngeal cavity, and aryepiglottic mucosa, adhered by viscous purulent phlegm. The patient refused any treatment for unknown etiology.

**FIGURE 2:**
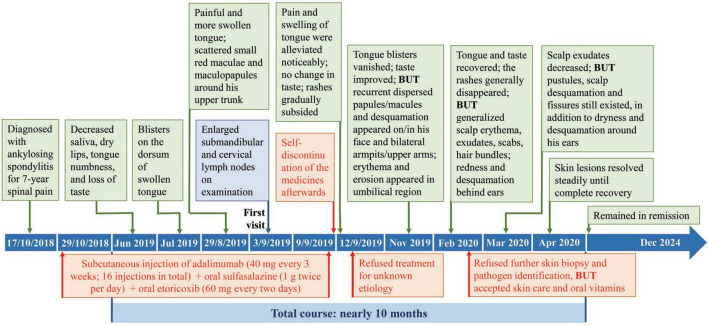
Disease progression after simultaneous use of adalimumab, sulfasalazine, and etoricoxib for ankylosing spondylitis.

During our first follow-up in November 2019, the superficial blisters on his tongue shrank or vanished, along with improvement in taste; however, recurrent dispersed papules/macules and desquamation appeared on/in his face and bilateral armpits/upper arms, accompanied by erythema and erosion in umbilical region (Figure 1). In February 2020, the patient consulted us again for generalized scalp erythema, exudates, scabs, hair bundles, redness and desquamation behind ears without pain/fever in recently 2 weeks, although the tongue and taste mostly recovered and the rashes in/on bilateral armpits/upper arms generally disappeared (Figure 1). Imaging examination of the skin lesions on his head and trunk demonstrated dotted and annular vascular distributions and yellow pustules under red background of skin lesions, with slight fluorescence under Wood’s lamp. The patient refused further skin biopsy and pathogen identification, but accepted skin care and oral vitamins. In March 2020, the scalp exudates decreased, but there were still pustules, scalp desquamation and fissures, in addition to dryness and desquamation around his ears (Figure 1). In April 2020, the skin lesions resolved steadily until complete recovery. He remained in remission during 4 years follow-up. The total course of disease was nearly 10 months (Figure 2). Written informed consent was obtained from the patient. This study was approved by the Ethics Committee of Shantou Longhu People’s Hospital.

## 3 Discussion

To the best of our knowledge, there is no report of an ensemble of strawberry tongue, dysgeusia, and similar systemic skin lesions in the literature. Therefore, this unusual case needs careful differentiation of a variety of conditions/diseases, including infections, allergy, immune diseases, tumors, and adverse drug reactions.

Strawberry tongue is mostly a result of bacterial toxin-mediated disorders (e.g., scarlet fever, recurrent toxin-mediated perianal erythema, and recalcitrant erythematous desquamating disorder), in addition to viral infection [e.g., yellow fever and coronavirus disease-2019 (COVID-19)] and non-infectious disorders (e.g., Kawasaki disease and food/drug allergies) (4, 5). The manifestations of chronic afebrile generalized papules/macules, pustules, and desquamation (no perianal erythema or desquamation) in our adult patient with no food allergies, normal vital signs and antistreptolysin O titer before the outbreak of COVID-19 do not support a diagnosis of the abovementioned diseases except for adverse drug reaction (differential diagnosis shown in Table 1).

**TABLE 1:** Differential diagnosis of adverse drug events caused by simultaneous use of adalimumab, sulfasalazine, and etoricoxib for ankylosing spondylitis.

Disease/condition	Supportive point	Unsupportive point	Conclusion
Coronavirus disease-2019	Strawberry tongue, dysgeusia, rashes	Onset before the outbreak of COVID-19	Excluded
Kawasaki disease	Strawberry tongue, dysgeusia, rashes, enlarged glands in the neck	Most common in children; no fever/chills or pains; normal heart ultrasound	Excluded
Food allergies	Strawberry tongue, dysgeusia, rashes	No food allergy history	Excluded
Scarlet fever	Strawberry tongue, rashes, enlarged glands in the neck	Most common in children; no fever/chills; normal vital signs and antistreptolysin O titer	Excluded
Yellow fever	Strawberry tongue, rashes	No travel history; no fever/chills, pains, fatigue, jaundice, bleeding, shock, or organ failure	Excluded
Recurrent toxin-mediated perianal erythema	Strawberry tongue, rashes	No perianal erythema or desquamation	Excluded
Recalcitrant erythematous desquamating disorder	Strawberry tongue, rashes	Recurrent and recalcitrant disorder with hypotension, tachycardia, and/or multiple-organ involvement	Excluded
Vitamin/mineral deficiencies	Dysgeusia, rashes	Normal serum folate, vitamin B12, and ferritin	Partly excluded
Diabetes	Dysgeusia, rashes	No diabetes history; normal serum glucose	Excluded
Hypothyroidism	Dysgeusia, rashes	Normal serum thyroid functions	Excluded
Kidney disease	Dysgeusia, rashes	No history of kidney disease; normal renal functions and urinary ultrasound	Excluded
Psoriasis	Dysgeusia, rashes	Chronic/recurring disease with multi-system involvement and multi-comorbidities	Excluded
Parapsoriasis	Rashes	Chronic/refractory condition mostly in the elderly without pustules	Excluded
Lichen psoriasis	Rashes	Apparent pruritus; usually no scalp involvement; permanent hair loss if scalp is affected	Excluded
Atopic dermatitis	Rashes	Chronic disease occurring since childhood with dry skin, severe pruritus, allergy history, and elevated serum Ig E	Excluded
Pityriasis rosea	Rashes	Acute and self-limited disease mostly in children and adolescents without pustules	Excluded

Dysgeusia can be caused by various factors, including aging, medications (e.g., immunosuppressants), high alcohol consumption, tobacco use, bacterial/viral/fungal infections (e.g., COVID-19), vitamin/mineral deficiencies, nerve damage, neurologic disorders (e.g., Alzheimer’s disease), diabetes, hypothyroidism, and kidney disease (6, 7). However, certain treatments or medications are usually the predominant cause of dysgeusia (7) and the highly suspected cause of dysgeusia in this case as well, since most of the other causes have been ruled out (Table 1).

Similarly, the skin lesions should be differentiated from varied dermatological diseases. The self-limited feature of generalized rashes with strawberry tongue and dysgeusia in our case is not consistent with psoriasis, parapsoriasis, lichen psoriasis, atopic dermatitis, and pityriasis rosea (Table 1).

Tumor necrosis factor blockers have been reported to induce paradoxically inflammatory psoriasis and/or lichen psoriasis (1, 3). Correspondingly, sulfasalazine as an anti-rheumatic agent and etoricoxib as a cyclooxygenase-2 inhibitor have similar profiles of adverse drug reactions, including rashes, serious infections, and hypersensitivity reactions (e.g., Stevens-Johnson syndrome, vasculitis, and parapsoriasis) (8, 9). Considering their pharmacological mechanisms, the reported similar adverse drug reactions, and gradual recovery and complete remission after cessation of the medicines, the triad-drug therapy might have triggered immunosuppression, opportunistic respiratory infection of *Acinetobacter pittii*, and the adverse drug events as dysgeusia, strawberry tongue, and psoriatic eruptions. The natural course of the disease without aggressive interventions subject to the willingness of the patient has provided us an invaluable opportunity to understand the dynamic changes of the adverse drug reactions. Administrations of intravenous immunoglobulins and/or topical treatment (corticosteroids, keratolytics, and vitamin D analogs) may help with alleviation of symptoms (1, 10).

## 4 Conclusion

This report has illuminated an unusual case of dysgeusia, strawberry tongue, and psoriasis-like eruptions after combination treatment with adalimumab, sulfasalazine, and etoricoxib for ankylosing spondylitis. Clinicians should be alert to the adverse drug reactions caused by the combination treatment with adalimumab, sulfasalazine, and etoricoxib. Rational use of TNF blockers as well as anti-inflammatory medicines is advocated.

## Data Availability

The original contributions presented in this study are included in this article/Supplementary material, further inquiries can be directed to the corresponding authors.
