# Supplementary material for: Case report: dysgeusia, strawberry tongue, and psoriatic eruptions after combination treatment with adalimumab, sulfasalazine, and etoricoxib for ankylosing spondylitis
Source: Front Med (Lausanne). 2025 Feb 3;12:1419922. doi: 10.3389/fmed.2025.1419922 (PMC11830582; doi:10.3389/fmed.2025.1419922)
Supplement: Supplementary file 1 [file Supplementary_file_1.docx]

**Supplementary Materials**

**Additional File 1.** Laboratory details of the patient with ankylosing spondylitis before and after combination treatment with adalimumab, sulfasalazine, and etoricoxib

| **Variable** | **Before the onset^a^** | | | **After the onset^a^** | | | **Reference range** |
| --- | --- | --- | --- | --- | --- | --- | --- |
|  | **8 months^b^** | **6 months** | **2 months** | **3 months** | **34 months** | **51 months** |  |
| ***Complete blood count*** | | | | | | | |
| White blood cell (10^9^ cells/L) | 8.98 | 5.76 | 9.02 | ***9.90*** | 7.66 | 8.80 | 3.5-9.5 |
| Neutrophils (10^9^ cells/L) | 6.22 | 2.99 | 5.49 | 6.20 | 5.10 | ***6.33*** | 1.8-6.3 |
| Lymphocytes (10^9^ cells/L) | 1.71 | 1.99 | 2.50 | 2.30 | 1.90 | 1.77 | 1.1-3.2 |
| Monocytes (10^9^ cells/L) | ***0.88*** | 0.57 | ***0.91*** | ***1.15*** | 0.50 | ***0.64*** | 0.1-0.6 |
| Red blood cell (10^12^ cells/L) | 5.00 | 5.08 | 4.77 | 4.53 | 5.06 | 4.89 | 4.3-5.8 |
| Hemoglobin (g/L) | 145 | 152 | 151 | 149 | 153 | 150 | 130-175 |
| Platelets (10^9^ cells/L) | 310 | 239 | 251 | 240 | 337 | 290 | 125-350 |
| ***Urinalysis*** | | | | | | | |
| Ketone body (mmol/L) | - | - | - | ***+*** | Negative | - | Negative |
| Leukocyte (cell/uL) | - | - | - | ***+/-*** | Negative | - | Negative |
| White blood cell (cells/L) | - | - | - | 7 | - | - | 0-7 |
| ***Fecal occult blood test*** | - | - | - | - | ***2+*** | Negative | Negative |
| ***C reactive protein (mg/L)*** | ***19.00*** | <0.80 | 2.11 | ***13.10*** | 3.88 | 4.60 | 0-8 |
| ***ESR (mm/hour)*** | ***23*** | 1 | 3 | ***34*** | - | - | 0-15 |
| ***Biochemical analysis*** | | | | | | | |
| Aspartate transaminase (U/L) | - | 22.0 | 21.0 | 16.0 | 19.0 | ***14.7*** | 15-40 |
| Alanine aminotransferase (U/L) | - | 37.0 | 32.0 | 9.9 | 15.0 | 19.8 | 9-50 |
| Total protein (g/L) | - | 70.88 | 71.93 | 73.51 | 73.20 | 72.00 | 65-85 |
| Albumin (g/L) | - | 42.86 | 45.29 | 44.49 | 41.50 | 44.45 | 40-55 |
| Globulin (g/L) | - | 28.02 | 26.64 | 29.02 | 31.70 | 27.55 | 20-40 |
| Uric acid (umol/L) | - | 398.7 | 404.3 | ***466.0*** | ***471.0*** | ***495.0*** | 210-430 |
| Blood urea nitrogen (mmol/L) | - | 5.74 | 5.01 | ***2.22*** | 4.70 | 4.64 | 2.50-7.14 |
| Creatinine (umol/L) | - | 86.00 | 84.00 | 73.00 | 70.50 | 83.32 | 44-115 |
| ***Anemia screening*** | | | | | | | |
| Folate (ng/ml) | - | - | 11.84 | 13.19 | - | - | >3.2 |
| Vitamin B12 (pg/ml) | - | - | ***992.65*** | ***109.36*** | - | - | 180-916 |
| Ferritin (ng/ml) | - | - | ***488.36*** | ***444.51*** | - | ***588.86*** | 27-375 |
| ***Pathogen testing*** | | | | | | | |
| HBsAg (IU/ml) | <0.03 |  |  |  | <0.03 |  | 0-0.05 |
| HCV antibody (S/CO) | - | - | - | Negative | Negative | - | Negative |
| HIV antibody (S/CO) | - | - | - | 0.06 | 0.10 | - | <1 |
| TPPA (S/CO) | - | - | - | Negative | Negative | - | Negative |
| TRUST (S/CO) | - | - | - | Negative | - | - | Negative |
| *Mycoplasma pneumonia* IgM | - | - | - | Negative | - | - | Negative |
| *Mycobacterium tuberculosis* DNA^c^ | - | - | - | Negative | - | - | Negative |
| EB-VCA IgA (s.c.o) | - | - | - | 0.63 | - | - | <1 |
| EB-VEA IgA (s.c.o) | - | - | - | ***1.86*** | - | - | <1 |
| EBV-DNA (copies/ml) | - | - | - | <500 | - | - | <500 |
| ***Tumor markers*** | | | | | | | |
| Alpha-fetoprotein (IU/ml) | - | - | - | 1.80 | 2.76 | 3.42 | 0-8.1 |
| CA-125 (U/ml) | - | - | - | 29.90 | - | - | 0-35 |
| CA-153 (U/ml) | - | - | - | 3.20 | - | - | 0-32.4 |
| CA-199 (U/ml) | - | - | - | 26.62 | 5.99 | - | 0-37 |
| Carcinoembryonic antigen (ng/ml) | - | - | - | <0.50 | 0.70 | <1.73 | 0-5 |
| ***Thyroid function*** | | | | | | | |
| TSH (mIU/L) | - | - | - | 2.34 | 2.16 | - | 0.55-5.5 |
| FT3 (pmol/L) | - | - | - | 4.11 | 4.89 | - | 3.5-6.5 |
| FT4 (pmol/L) | - | - | - | 14.54 | 11.44 | - | 7.9-22.7 |
| ***HLA-B27 (%)*** | ***97.10*** | - | - | - | - | - | <80 |
| ***Antistreptolysin (IU/ml)*** | <25.0 | - | - | <200 | - | - | <200 |
| ***Rheumatoid factor (IU/ml)*** | <20.0 | - | - | - | - | - | 0-20 |
| ***Immunoglobulin*** | | | | | | | |
| IgM (g/L) | 1.31 | - | 1.25 | 2.21 | - | 1.05 | 0.46-3.04 |
| IgG (g/L) | 12.20 | - | 12.70 | 15.50 | - | 13.90 | 7.51-15.6 |
| IgA (g/L) | 2.16 | - | 2.30 | 3.73 | - | 3.52 | 0.82-4.53 |
| IgE (IU/ml) | - | - | - | - | 55.21 | - | 10.0-90.0 |
| C3 (g/L) | 1.12 | - | ***0.78*** | 0.93 | - | 0.97 | 0.79-1.52 |
| C4 (g/L) | 0.28 | - | 0.17 | 0.20 | - | 0.27 | 0.16-0.38 |
| ***Immunological test (antibodies)*** | | | | | | | |
| Anti-U1RNP/Sm | - | - | - | Negative | - | - | Negative |
| Sm | - | - | - | Negative | - | - | Negative |
| SS-A | - | - | - | Negative | - | - | Negative |
| SS-B | - | - | - | Negative | - | - | Negative |
| Scl-70 | - | - | - | Negative | - | - | Negative |
| Jo-1 | - | - | - | Negative | - | - | Negative |
| Anti-ribosomal phosphoprotein | - | - | - | Negative | - | - | Negative |
| Ro-52 | - | - | - | Negative | - | - | Negative |
| Centromere protein B | - | - | - | Negative | - | - | Negative |
| dsDNA | - | - | - | Negative | - | - | Negative |
| Anti-nucleosome antibodies | - | - | - | Negative | - | - | Negative |
| Anti-histone antibodies | - | - | - | Negative | - | - | Negative |
| ***Lymphocyte subset*** |  |  |  |  |  |  |  |
| CD3+ (%) | - | - | - | 58.00 | - | 51.39 | 50-84 |
| CD45+CD3+CD4+ (%) | - | - | - | ***25.97*** | - | ***25.87*** | 27-51 |
| CD45+CD3+CD8+ (%) | - | - | - | 27.72 | - | 19.11 | 15-44 |
| CD4+/CD8+ | - | - | - | ***0.94*** | - | 1.35 | 0.98-1.94 |
| CD3+ (cells/μL) | - | - | - | 1,038.20 | - | - | 955-2,860 |
| CD45+CD3+CD4+ (cells/μL) | - | - | - | ***464.86*** | - | ***-*** | 550-1,440 |
| CD45+CD3+CD8+ (cells/μL) | - | - | - | 496.19 | - | - | 320-1,250 |

Abnormal values are bolded and italicized. ESR, erythrocyte sedimentation rate; HBsAg, hepatitis B surface antigen; HIV, human immunodeficiency virus; TPPA, treponema pallidum particle agglutination; TRUST: toluidine red unheated serum test; HCV, hepatitis C virus; EB-VCA, Epstein-Barr viral capsid antigen; EB-VEA, Epstein-Barr virus early antibody; EBV, Epstein-Barr virus; SARS-CoV-2, severe acute respiratory syndrome-coronavirus-2; TSH, thyroid stimulating hormone; FT3, free triiodothyronine; FT4, free thyroxine; HLA-B27, human leukocyte antigen B27; CD3+, T cells; CD45+CD3+CD4+, helper-inducer T cells; CD45+CD3+CD8+, suppressor-killer T cells

^a^Onset of dysgeusia (i.e., eight months after treatment with adalimumab, sulfasalazine, and etoricoxib)

^b^Baseline profiles before treatment

^c^Collected from sputum
